# A Rare Renal Epithelial Tumor: Mucinous Cystadenocarcinoma Case Report and Review of the Literature

**DOI:** 10.1155/2011/686283

**Published:** 2011-10-29

**Authors:** Abdulkadir Tepeler, Mehmet Remzi Erdem, Omer Kurt, Ramazan Topaktas, Isin Kilicaslan, Abdullah Armağan, Şinasi Yavuz Önol

**Affiliations:** ^1^Department of Urology, Faculty of Medicine, Bezmialem Vakif University, Istanbul 34093, Turkey; ^2^Department of Urology, Bayrampasa State Hospital, Istanbul 34093, Turkey; ^3^Department of Pathology, Istanbul Medical Faculty, Istanbul University, Istanbul 34093, Turkey

## Abstract

Primary renal mucinous cystadenocarcinoma is a very rare lesion of kidney which originates from the metaplasia of the renal pelvic uroepithelium. Only one case with primary mucinous cystadenocarcinoma has been reported in the English literature. We report second case of mucinous cystadenocarcinoma which was radiologically classified as type-IIF Bosniak cyst in peripheral localization. We aimed to present this extreme and unusual entity with its radiological, surgical, and pathologic aspects under the light of literature.

## 1. Introduction

Although the most renal tumors originate from the kidney parenchyma, renal uroepithelium rarely hosts epithelial tumors. Mucinous cystadenocarcinoma (MC) comprises less than 1% of malignancies which arise from epithelium of the renal pelvis [1]. Primary mucinous epithelial tumors occurring in the kidney are presumed to originate from the metaplasia of pluripotent renal epithelial cells to glandular lesions [2]. Although they have mostly benign potential, only one case has been reported where the epithelial lining of the tumor was malign [3]. 

Because of the pelvic localization, mucinous epithelial tumors generally show signs as mucusuria, hematuria, and flank pain. However, no sign may be observed if the lesion locates peripherally. Moreover, benign lesions mimic simple renal cysts radiologically.

We describe a case with renal MC, peripherally located and diagnosed during evaluation of lower urinary tract symptoms which was radiologically defined as complicated renal cyst in light of the literature.

## 2. Case Report

**Figure 1:**
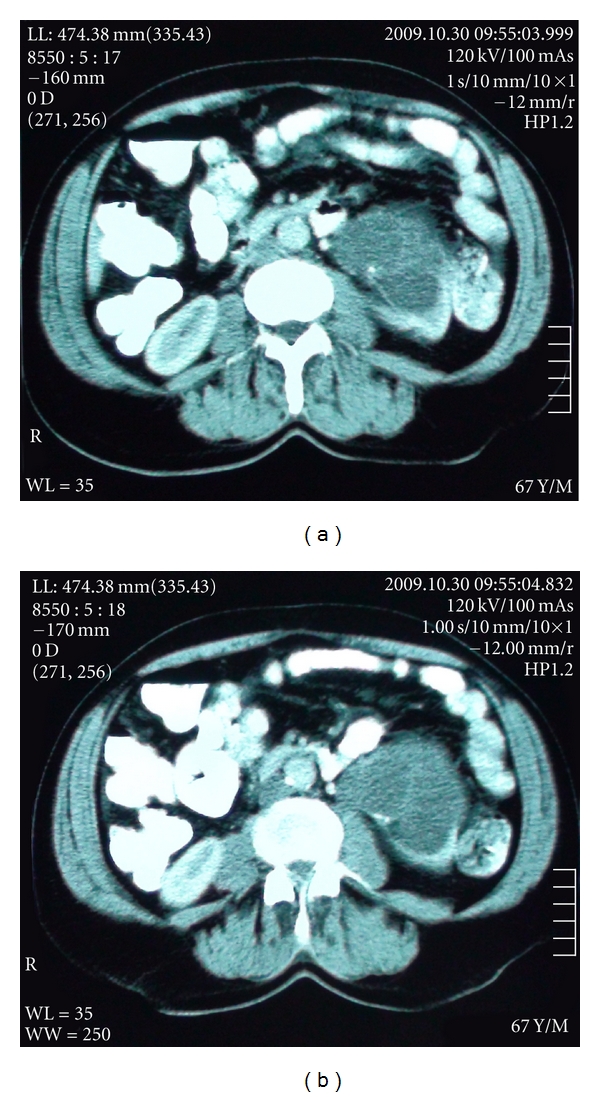
Cystic lesion, originated from renal pelvis, and containing multilocular cystic structure divided by contrasted septate and covered with calcified wall in 7 × 6 × 6 cm size (Bosniak-IIF), was displayed on contrast-enhanced computerized tomography.

A 60-year-old man presented with an incidental mass that had been firstly noticed for microscopic hematuria during evaluation of lower urinary tract symptoms. A hyperechoic cystic lesion was firstly detected with ultrasound at proximal to dilated left renal pelvis. Cystic lesion, originated from renal pelvis, containing multilocular cystic structure divided by contrasted septate, and covered with calcified wall in 7 × 6 × 6 cm size (Bosniak-IIF) was displayed on contrast-enhanced computerized tomography (Figure 1). 

Partial nephrectomy was planned because of suitable size in spite of location at expanding to the renal hilum. The inadvertent minimal opening on the cyst wall caused a change in the decision about operation plan as radical nephrectomy. We prevented any leakage from content of cyst during radical nephrectomy by clamping the two sides of tear on the wall.

**Figure 2:**
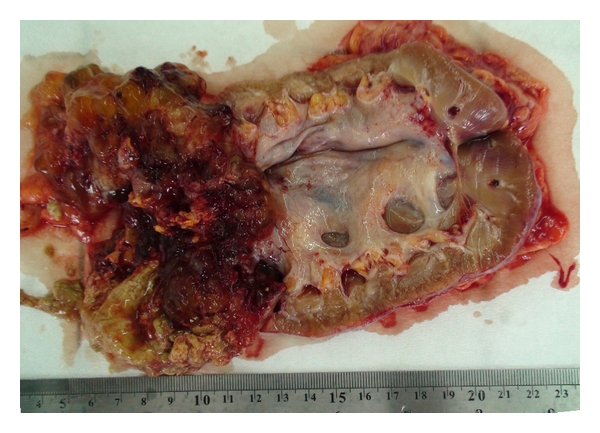
Grossly, a tumor which was 7 cm in size originated from renal pelvis and expanded to the upper pole. The mass was filled with chocolate color mucinous fluid.

Grossly, the tumor was a multilocular cystic mass filled with chocolate color mucinous fluid (Figure 2). This morphologic appearance called to mind metastatic spread of ovarian or appendiceal mucinous neoplasia. Although this cyst had morphologically looked like mentioned possibility, patient's gender and medical history, who was male and appendectomised, were not proper to suggested theories. 

**Figure 3:**
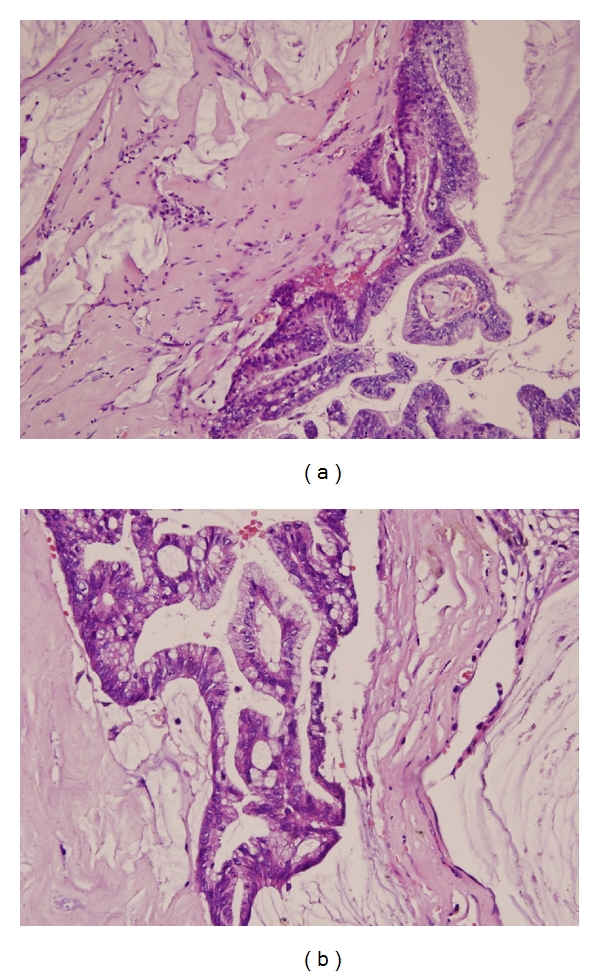
Adenoid structures formed by multilayer atypical epithelial cells and interstitial mucine lakes.

Microscopically, renal parenchymal thinning, multiloculated and mucoid content, and partially papillary structures were defined in this cystic lesion. Histologically, multilayered tall columnar mucinous epithelium forming the cyst wall and resembling intestinal and endocervical epithelium, was seen gaining gradual malignancy potential with formation of glands of different sizes and shapes lined by columnar cells having hyperchromatic and pleomorphic nuclei, and eosinophilic-to-vacuolated cytoplasm (Figure 3). Also nuclear atypia and stromal invasion which are characteristic features of malignant type of MC were obviously seen. Positive immunohistochemical staining with CEA, CK7, and EMA and negative staining of CK20 fortify diagnosis.

The patient did well postoperatively and his followup during 28 months after operation was troubleless. Contrast-enhanced abdominal CT and thorax X-ray film were negative for any local or metastatic invasion at postoperative 24th month.

## 3. Discussion

MC is an extremely rare tumor of the kidney. It originates from glandular metaplasia of the pluripotent uroepithelium lining the renal collector system. Although these tumors generally locate in the renal pelvis, different locations where the uroepithelium present such as bladder, ureter, and renal calyxes may host them. But only a few cases with atypical localization are reported in the literature [4, 5]. In the present case, the tumor was originated from upper pole but extending to the renal pelvis. 

Three theories are suggested about formation of glandular metaplasia (i) chronic irritation, (ii) differentiation of coelomic epithelium, and (iii) maldevelopment of kidney. Moreover, it has been thought that chronic irritation due to infection, urolithiasis, and uroepithelium which was exposed to high pressure causes metaplastic transformation of transitional epithelium on renal pelvis [6]. 

Renal mucinous cystadenomas behave as a large different clinical range from pseudomyxoma peritonei to silent cystic lesion without any complication [2, 7]. Although the most of the patients are asymptomatic, hematuria, mucusuria, flank pain, and palpable abdominal mass are the other clinical presentations [3]. 

Most of the renal tumors are radiologically diagnosed, and ultimate decision for treatment is generally made according to radiologic imaging. The positive predictive value of radiologic imaging is so high that a negative biopsy result does not alter management [8]. However, prediction of malignancy potential of renal complex cyst and to decide whether there is a necessity of surgical exploration or not with only radiologic images is quite difficult. Furthermore, mucinous cysts may present themselves in different types of Bosniak classifications. Akan et al. reported a mucinous cystadenoma mimicking simple renal parenchymal cyst radiologically [7]. In the present case, the mass was reported as type-IIF renal cyst because of the contrasted septate and calcified wall. Type-IIF is a new transitional zone between suspected benign and malignant lesions. They must be strictly followed because of high malignancy potential.

The exact diagnosis is made after the pathologic examination. Pathologic appearance defines the malignancy, according to stromal invasion, nuclear atypia, and multilayers of neoplastic cells. Especially stromal invasion is the definitive marker of malignancy. There is a confusion of nomenclature of this extremely rare tumor. Renal mucinous cystadenoma and the renal MC are different entities, and they commonly confound with the mucinous adenocarcinoma and papillary cystadenocarcinoma. In light of this pathological knowledge, most of the mucinous cysts are defined as benign; there are 3 cases with borderline characteristics and only two cases with malignant transformation [2, 5, 9–11]. There is only one case with primary mucinous cystadenocarcinoma of the renal pelvis recently published [3]. 

Surgical excision of the mass is a sufficient treatment method for these cases. Radical or simple nephrectomy has been performed for the previous cases. Surgeons should be careful while dissecting the cyst because of the perforation of the cyst wall and risk of tumor spreading. In the present case, we decided to perform radical procedure due to cyst wall perforation and inability to dissect the whole cyst. However we took all measures to prevent the tumor spreading. Only one case was presented with disseminated pseudomyxoma peritonei after one year following the radical nephrectomy [2]. If definitive therapy will be radical nephrectomy, additional ureterectomy should be performed especially in cases presented with mucusuria like the transitional cell carcinoma of the renal pelvis, because implantation of tumor cells by mucusuria is still unknown.

Finally, complex renal cystic lesions should be carefully followed for early detection of malignancy. Because management of these uncommon lesions is not clearly defined, radical or partial nephrectomy should be the first-line therapy and second steps must be planned under guidance of pathology results. On the other hand, mucinous cystadenoma must be classified into the renal malignancy and confusion of nomenclature must be corrected for preventing misapplication.
